# Cytokine release syndrome

**DOI:** 10.1186/s40425-018-0343-9

**Published:** 2018-06-15

**Authors:** Alexander Shimabukuro-Vornhagen, Philipp Gödel, Marion Subklewe, Hans Joachim Stemmler, Hans Anton Schlößer, Max Schlaak, Matthias Kochanek, Boris Böll, Michael S. von Bergwelt-Baildon

**Affiliations:** 1 Cologne Interventional Immunology, University Hospital of Cologne, Cologne, Germany; 2 Intensive Care Program, Department I of Internal Medicine, University Hospital of Cologne, Cologne, Germany; 3 Center of Integrated Oncology Cologne-Bonn, University Hospital of Cologne, Cologne, Germany; 4 Intensive Care in Hemato-Oncologic Patients (iCHOP), Cologne, Germany; 5 Department of Medicine III, University Hospital, LMU Munich, Munich, Germany; 6 Translational Cancer Immunology, Gene Centre, University of Munich, Munich, Germany; 7 German Cancer Consortium (DKTK), Heidelberg, Germany; 8 Department of General, Visceral and Cancer Surgery, University Hospital of Cologne, Cologne, Germany; 9 Department of Dermatology/Venereology, University Hospital of Cologne, Cologne, Germany; 10 Comprehensive Cancer Center Munich (CCCM), Munich, Germany

**Keywords:** Cytokine release syndrome, Immunotherapy, CAR T cells, T cell-engaging therapies, Cytokine storm

## Abstract

During the last decade the field of cancer immunotherapy has witnessed impressive progress. Highly effective immunotherapies such as immune checkpoint inhibition, and T-cell engaging therapies like bispecific T-cell engaging (BiTE) single-chain antibody constructs and chimeric antigen receptor (CAR) T cells have shown remarkable efficacy in clinical trials and some of these agents have already received regulatory approval. However, along with growing experience in the clinical application of these potent immunotherapeutic agents comes the increasing awareness of their inherent and potentially fatal adverse effects, most notably the cytokine release syndrome (CRS). This review provides a comprehensive overview of the mechanisms underlying CRS pathophysiology, risk factors, clinical presentation, differential diagnoses, and prognostic factors. In addition, based on the current evidence we give practical guidance to the management of the cytokine release syndrome.

## Background

Cytokine release syndrome (CRS) is a systemic inflammatory response that can be triggered by a variety of factors such as infections and certain drugs. The term “cytokine release syndrome” was first coined in the early ‘90s, when the anti-T-cell antibody muromonab-CD3 (OKT3) [1, 2] was introduced into the clinic as an immunosuppressive treatment for solid organ transplantation. Subsequently, CRS has been described after infusion of several antibody-based therapies such as anti-thymocyte globulin (ATG) [3], the CD28 superagonist TGN1412 [4], rituximab [5], obinutuzumab [6], alemtuzumab [7], brentuximab [8], dacetuzumab [9], and nivolumab [10]. CRS has also been observed following administration of non-protein-based cancer drugs such as oxaliplatin [11] and lenalidomide [12]. Furthermore, CRS was reported in the setting of haploidentical donor stem cell transplantation, and graft-versus-host disease [13, 14]. Cytokine storm due to massive T cell stimulation is also a proposed pathomechanism of severe viral infections such as influenza [15, 16].

Lately, with the success of the newer T cell-engaging immunotherapeutic agents there has been a growing interest in CRS since it represents one of the most frequent serious adverse effects of these therapies. T cell-engaging immunotherapies include bispecific antibody constructs and chimeric antigen receptor (CAR) T cell therapies. Both these immunotherapeutic strategies have recently been carried forward into clinical application and have shown impressive therapeutic activity in several hematologic malignancies, such as acute lymphoblastic B cell leukemia (B-ALL), chronic lymphocytic leukemia (CLL), and diffuse large B cell lymphoma (DLBCL).

In 2014, the CD19-directed CD3 BiTE blinatumomab was approved for Philadelphia chromosome-negative relapsed or refractory B-cell precursor ALL under the FDA’s accelerated approval program [17]. Recently, the first two CAR T cell therapies tisagenlecleucel and axicabtagene ciloleucel received FDA approval for refractory CD19-positive B-ALL [18] and relapsed or refractory large B-cell lymphoma [19]. Multiple other bispecific antibody and CAR T cell constructs that target a variety of antigens are currently in clinical development. Furthermore, there are a number of related T cell-engaging immunotherapeutic approaches in earlier clinical development. These include dual-affinity re-targeting antibodies (DART), immune-mobilising monoclonal TCRs against cancer (ImmTAC), and other TCR-based strategies [20, 21].

Studies of the first T cell-engaging therapies, i.e. blinatumomab [22] and CD19-targeted CAR T cells [23–25] revealed that CRS is the most important adverse event of these therapies. Thus, most of the current CRS data is derived from CAR T cell and blinatumomab studies in hematologic malignancies where CRS has been reported in frequencies of up to 100% in CD19-targeted CAR T cell trials, sometimes with fatal outcome (Table 1). As in the future, T cell-engaging immunotherapeutic agents will increasingly be used outside of clinical studies and academic cancer centers it becomes paramount that oncologists and intensive care specialists are familiar with this complication and its clinical management.

**Table 1:** CRS reported in recent clinical trials

Author/Year	Maude et al., 2018 [87]00	Park et al., 2018 [88] 00	Neelapu et al., 2017 [44] 00	Schuster et al., 2017 [89] 00	Turle et al., 2017	Gardner et al., 2017 [80] 8]	Ali et al., 2016 [79] 7]	Garfall et al., 2015 [90] 3]	Lee et al., 2015 [39] 5]	Maude et al., 2015 [32] 8]	Davila et al., 2014 [33] 9]	Kantarjian et al., 2017 [27] 4]	Stackelberg et al., 2016 [91] 5]	Topp et al., 2015 [68] 7]	Topp et al., 2014 [92] 6]
Institution	25 centers	MSKCC	22 centers	UPenn	FHCRC	SCHRI	NCI	UPenn	NCI	UPenn/UPhil	MSKCC	101 centers	26 centers	37 centers	9 centers
Applied therapy	CD19 CAR (4-1BB)	CD19 CAR (CD28)	CD19 CAR (CD28)	CD19 CAR (4-1BB)	CD19 CAR (4-1BB)	CD19 CAR (4-1BB)	BCMA CAR (CD28)	CD19 CAR (4-1BB)	CD19 CAR (CD28)	CD19 CAR (4-1BB)	CD19 CAR (CD28)	blinatumomab	blinatumomab	blinatumomab	blinatumomab
Disease	B-ALL	B-ALL	DLBCL/TFL/ PMBCL	DLBCL/TFL	CLL	B-ALL	MM	MM	B-ALL	B-ALL	B-ALL	B-ALL	B-ALL	B-ALL	B-ALL
Number of patients	75	53	101	28	24	45	12	11	20	30	16	267	70	189	36
Incidences															
% CRS	77	85	93	57	83	93	50^1^	18	66	100	NR	14,2	11	NR	NR
% sCRS (>°II)	46	26	13	18	8	23	17^1^	9	32	27	44	4,9	5	3	0,8
% sNeurotox (>°II)	13	42	28	11	25	21	0	NR	1	3	13	9,4	4	11	14
treatment related deaths	1^2^	1^3^	3^4^	1^5^	1^6^	0	0	0	0	0	0	3^7^	6^8^	1^9^	3^10^
sCRS correlates															
tumor burden	NR	y*	NR	NR	y	n	NR	NR	y	y	y	NR	NR	NR	y
CRP/ferritin	n/y	NR	NR	NR	y*/y*	NR	NR	NR	y*/y*	y*^11^/y*	y*/NR	NR	NR	NR	NR
IL-6/IFNg	y/y	NR	y/n	NR	y*/y*	y/NR	NR	NR	y*/y*	y*/y*	n*/n*	NR	NR	NR	NR
Therapy															
tocilizumab response	NR	NR	NR	1/1	5/6	NR	100	NR	50%^12^	100%	y	NR	NR	NR	NR
steroid response	NR	NR	NR	0/0	5/6	NR	NR	NR	y	y	y	NR	NR	NR	NR
Prognosis															
CRS related to ORR?	NR	NR	NR	NR	NR	NR	NR	NR	y	NR	NR	NR	NR	2/3 CR	y
tocilizumab related to reduced ORR?	NR	NR	n	n	NR	n	n	NR	n	possible^13^	n	NR	NR	NR	NR
steroids related to reduced ORR?	NR	NR	n	NR	NR	n	NR	NR	n	possible^13^	y	NR	NR	NR	NR
Response															
% ORR	81	83	82	64	71	100	25	NR	33	10	NR	NR	45	52	75
% CR (MRD -)	81 (81)	83 (67^14^)	55	57	17	93 (93)	8	NR	66 (62)	90	88 (75)	44	39 (20)	43 (32)	69 (61)

^1^: no definition of CRS supplied, but patients showed signs of CRS. ^2^: death due to cerebral hemorrhage in the context of coagulopathy and resolving cytokine release syndrome. ^3^: 1 death due to °V CRS before dose adjustment to disease burden. ^4^: 2 deaths attributed to CRS, 1 to pulmonary embolism. ^5^: 1 death due to neurotoxicity. ^6^:1 death to °V CRS with °V cerebral edema refractory to tocilizumab, siltuximab, dexamethasone before reduction of CAR T cell dose. ^7^: not further specified. ^8^: 6 fatal AEs attributed to blinatumomab, one due to °IV CRS with °V respiratory failure. ^9^: 1 death due to fungal infection of the brain after HSCT classified as possibly related to blinatumomab. ^10^: 3 deaths due to sepsis and candida infection classified as possibly related to blinatumomab. ^11^: CRP > 20 mg/dl: positive predictive value only 50%.^12^: 2 patients received steroids additional to tocilizumab for not reported reasons.^13^: 2 of 9 patients treated with immunosuppression (not further specified if related to tocilizumab or glucocorticoids) relapsed. ^14^: 48 patients evaluable. *: statistically significant

*B-ALL* acute lymphoblastic B cell leukemia, *CLL* chronic lymphocytic leukemia, *DLBCL* diffuse large B cell lymphoma, *MM* Multiple myeloma, *MPM* malignant pleural mesotheliomas, *NR* not reported, *PDA* pancreatic ductal adenocarcinoma, *PMBCL* primary mediastinal large B-cell lymphoma, *TFL* transformed follicular lymphoma, *sNeurotox* severe neurotoxicity

## Review

### Clinical presentation

**Fig. 1:**
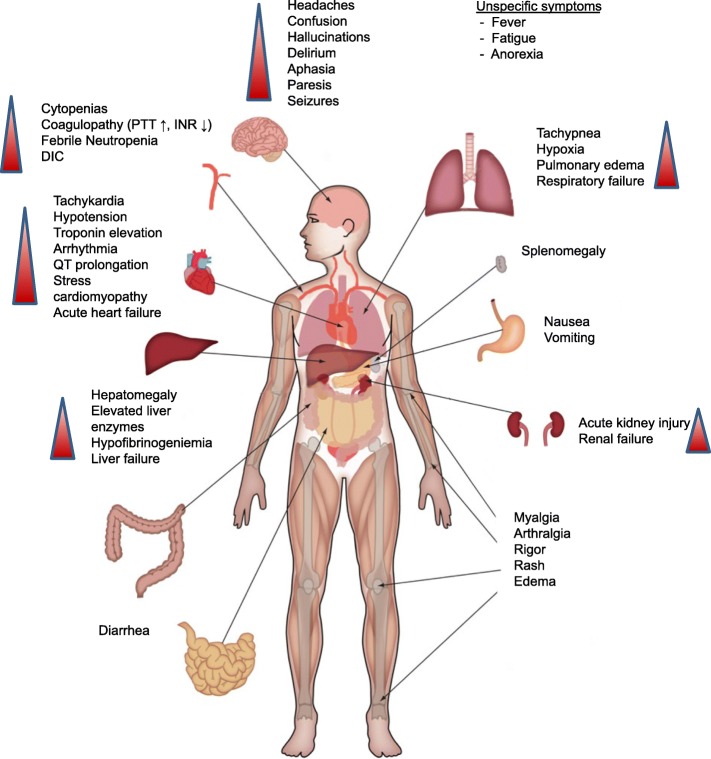
Clinical presentation of CRS. Beginning with fever and unspecific symptoms CRS might impact most organ systems. Mild cases can present as flu-like illness. Grade °III to IV shows signs of life threatening cardiovascular, pulmonary and renal involvement. Neurotoxicity can occur concurrent or with delay. Abbreviations: DIC: disseminated intravascular coagulation; INR: international normalized ratio; PTT: partial thromboplastin time

CRS can present with a variety of symptoms ranging from mild, flu-like symptoms to severe life-threatening manifestations of the overshooting inflammatory response (Fig. 1). Mild symptoms of CRS include fever, fatigue, headache, rash, arthralgia, and myalgia. More severe cases are characterized by hypotension as well as high fever and can progress to an uncontrolled systemic inflammatory response with vasopressor-requiring circulatory shock, vascular leakage, disseminated intravascular coagulation, and multi-organ system failure. Laboratory abnormalities that are common in patients with CRS include cytopenias, elevated creatinine and liver enzymes, deranged coagulation parameters, and a high CRP.

Respiratory symptoms are common in patients with CRS. Mild cases may display cough and tachypnea but can progress to acute respiratory distress syndrome (ARDS) with dyspnea, hypoxemia, and bilateral opacities on chest X-ray. ARDS may sometimes require mechanical ventilation. Of note, in patients with CRS the need for mechanical ventilation is oftentimes not due to respiratory distress but instead a consequence of the inability to protect the airway secondary to neurotoxicity [26]. Patients with severe CRS can also develop renal failure or signs of cardiac dysfunction with reduced ejection fraction on ultrasound. In addition, patients with severe CRS frequently display vascular leakage with peripheral and pulmonary edema.

In severe cases CRS can be accompanied by clinical signs and laboratory abnormalities that resemble hemophagocytic lymphohistiocytosis (HLH) or macrophage activation syndrome (MAS). Patients with CRS-associated HLH display the typical clinical and laboratory findings of HLH/MAS such as high fevers, highly elevated ferritin levels, and hypertriglyeridemia. In a phase III study of blinatumomab in B-ALL four out of 13 CRS patients showed signs of HLH [27].

Some patients develop neurotoxicity after administration of T cell-engaging therapies. Neurologic symptoms might span from mild confusion with word-finding difficulty, headaches and hallucinations to aphasia, hemiparesis, cranial nerve palsies, seizures and somnolence. In the case of CAR T cell therapy, neurotoxicity represents the second most common serious adverse event and therefore the term “CAR T cell-related encephalopathy syndrome” (CRES) has been introduced [28]. The neurotoxicity of CAR T cell therapy does not seem to be directly related to CRS since neurologic symptoms do not always coincide with CRS onset and neurotoxicity can occur prior to CRS or after CRS has resolved [29]. The pathophysiology of the neurologic symptoms is poorly understood, but the lack of a strict temporal association with CRS indicates that it might, at least in part, be independent from CRS. In addition, experience from clinical trials suggests that treatment of the neurologic symptoms is different from that of CRS.

### Epidemiology

The incidence of CRS in patients receiving cancer immunotherapy varies widely depending on the type of immunotherapeutic agent. The onset of CRS can occur within a few days, and in the case of CAR T cell therapy, up to several weeks after infusion of the drug. With most conventional monoclonal antibodies the incidence of CRS is relatively low, whereas T cell-engaging cancer immunotherapies carry a particularly high risk of CRS.

Although most responding patients experience at least some degree of CRS there seems to be no direct association between the severity of CRS and clinical response. CRS does not seem to be a prerequisite for response to T cell-engaging therapies. Some patients show complete remission without obvious signs of CRS, while other patients display severe symptoms and laboratory abnormalities but no clinical response.

Clinical studies identified a number of predictors of CRS severity. The risk of CRS is influenced by factors related to the type of therapy, the underlying disease, and characteristics of the patients. Several clinical factors are associated with the severity of CRS following CAR T-cell therapy. Many CRS-inducing agents display a “first-dose effect”, i.e. the most severe symptoms only occur after the first administered dose and do not recur after the subsequent administrations [30]. This “first-dose effect” is thought to be due to the higher disease burden at initiation of treatment. Disease burden is among the most important predictors of severe CRS after CAR T cell therapy or bispecific T cell-engager administration [5, 31–35]. For instance, in patients with ALL the burden of disease was associated with the severity of CRS [36]. Similar observations have been made in a murine lymphoma model, were injection of CAR T cells into mice with high tumor burden resulted in lethal CRS, whereas mice with low tumor burden did not show signs of CRS [37, 38].

The administered dose of the active agent is another factor that affects the risk of CRS [33, 35, 39]. Furthermore, the strength of T cell activation and the degree of T cell expansion seem to correlate with the severity of CRS [40]. Children seem to be at a higher risk of developing CRS than adults. In pediatric patients with ALL the incidence of CRS following infusion of CD19-targeted CAR T cells was 30/30 (100%) and 16/21 (76%) in two clinical trials testing distinct 19-targeted CAR constructs [39, 41]. The causes of the higher incidence of CRS in pediatric patients are unknown but may be related to higher cell dose used or the more immature immune system of children.

The type of T cell-engaging agent affects the overall risk as well as the onset of CRS. Even though the activation of T cells is the common underlying trigger in all types of T cell-engaging therapies, there are also important differences between the different therapeutic agents that affect the incidence, time course, and clinical management of CRS. Since CAR T cells can persist in the circulation for more than 1 year, the risk for CRS extends for a longer period of time but generally is highest up to 2 weeks after infusion. In CAR T cell therapy the nature of the CAR construct influences the likelihood, severity and time to clinical manifestation of CRS. Whereas CRS was rarely observed in studies of first generation CAR T cell constructs that lacked additional costimulatory signaling domains, CRS is much more commonly reported with second generation CAR constructs [42]. Even among the different second generation CARs there are differences in the rate of CRS. CARs with the CD28 costimulatory domain induce a brisk but self-limited CAR T cell expansion whereas the 4-1BB costimulatory domain promotes longer persistence [43]. CARs that incorporate a CD28 costimulatory domain seem to be associated with a higher risk of CRS. In two randomized trials of CAR T cells in patients with NHL the incidence of CRS was 93% with a CD28-containing CAR and 57% with a 4-1BB-containing CAR [44, 45]. However, due to differences in the patient populations and differences in the definition of CRS, no definitive conclusions can be drawn with regard to CAR design and the associated risk of CRS. Finally, the type of lymphodepletion that was used prior to CAR T cell infusion affected the risk of CRS. A higher incidence of CRS was observed after lymphodepletion with cyclophosphamide or fludarabine [26]. This was most likely a consequence of the higher expansion rates secondary to the more pronounced lymphodepletion achieved by combination therapy.

### Differential diagnoses

Clinically, CRS patients present with unspecific syndromes making the diagnosis challenging. It is important to distinguish CRS from other inflammatory disorders that present with similar clinical signs and symptoms but require different treatment.

Tumor lysis syndrome (TLS) can mimic CRS and presents with symptoms such as fever, acute renal failure, cardiac arrhythmia, and seizures. Although tumor lysis syndrome usually can be readily discriminated from CRS on the basis of characteristic laboratory abnormalities such as hyperuricemia, hyperkalemia, hyperphosphatemia and hypocalcemia, it can sometimes be difficult to determine if CRS and tumor lysis syndrome occur concurrently [46].

It is important to distinguish patients with CRS from those with sepsis since the treatment for CRS could be detrimental if used in patients with sepsis. Unfortunately, it is extremely difficult to distinguish sepsis from CRS. In fact, according to the most recent definition a large percentage of patients with severe CRS will fulfill the clinical criteria of sepsis, i.e. suspected infection with organ dysfunction defined as an increase of 2 points or more in the Sequential Organ Failure Assessment (SOFA) score [47]. Furthermore, a significant proportion of these patients will also fulfill the criteria for septic shock since they have an elevated lactate and require vasopressors.

Patients with CRS are at a high risk of infection and the immunosuppressive treatment that is administered for the treatment of CRS can mask some of the signs of infection thereby delaying diagnosis and treatment of infection. In one study of 133 patients receiving CD19-targeted CAR T cell therapy, 23% of patients developed infection within the first 4 weeks after CAR T cell infusion [48]. The infections typically began after the onset of CRS. Among the 93 patients with CRS, 28 (30%) developed an infection.

Infections that occur in patients with CRS are predominantly of bacterial origin, followed by viral infections that primarily involve the respiratory tract. Fungal infections are rare and were primarily observed in patients that had previously undergone autologous or allogeneic stem cell transplantation and were suffering from severe CRS [48]. The majority of infections occurred early after CAR T cell infusion and CRS severity was the most important risk factor for infection. It therefore is crucial to maintain a high degree of vigilance for infection and appropriate empiric antimicrobial therapy should be rapidly initiated if infection is suspected. All patients with CRS should receive an extensive diagnostic work-up to exclude infections, including a chest X-ray and blood cultures. Furthermore, before start of immunotherapy patients should be carefully checked for any signs of infection [49]. The mechanism that is responsible for the increased incidence of infection in patients with CRS is unknown. The CRS-associated propensity for infections resembles the severe immunosuppression in patients HLH/MAS, which also are at a high risk of serious infectious complications. A plausible explanation could be that the massive release of cytokines in CRS induces a form of immune paralysis, which predisposes the patients to an increased risk of infection. This hypothesis is consistent with the observation that the incidence of infections is higher in patients with more severe CRS [48].

As already mentioned, a HLH/MAS-like syndrome can develop as part of the CRS and usually is a manifestation of severe CRS. CRS-related HLH is difficult to distinguish from primary HLH or other conditions that can mimic HLH such as sepsis. Table 2 summarizes some of the factors that help to distinguish CRS-related HLH from other conditions that present similarly. Even though in most cases HLH/MAS that develops concurrently with CRS is triggered by CRS, other causes of HLH/MAS, such as genetic defects (in pediatric patients), autoimmune disease, infection, or the underlying malignancy itself should be taken into account. Patients with severe neurotoxicity require a thorough neurologic work-up which should include a careful neurologic exam and, if appropriate, brain imaging, a spinal tab and an electroencephalogram.

**Table 2:** Differential diagnoses of CRS-related HLH/MAS

	Familial HLH	Secondary HLH/MAS	CRS-related HLH/MAS	Sepsis
Genetic Predisposition	Homozygous mutations	Heterozygous mutations in some patients	unknown	unknown
Age group	Young children	All ages	All ages	All ages
Biomarkers				
IL-10	↑↑↑	↑↑↑	↑	↑
IFN-γ	↑↑↑	↑↑↑	↑↑↑	←→
IL-6	↑	↑	↑↑↑	↑↑↑
Ferritin	↑↑↑	↑↑↑	↑↑↑	↑
CD163	↑↑↑	↑↑↑	NDA	↑

*CRS* cytokine release syndrome, *HLH* hemophagocytic lymphohistiocytosis, *MAS* macrophage activation syndrome, Sepsis. *NDA* no data available

Since most T cell-engaging agents contain non-human protein sequences there is a risk of allergic drug reactions. Hypersensitivity reactions can also present with rash and urticaria, fever, dyspnea, hypotension and gastrointestinal symptoms culminating in cardiorespiratory failure. Unlike in CRS symptoms of true type I reactions occur after repeated exposure to the causative agent [50, 51]. Physicians should consider allergic reactions as a cause for the patients’ symptoms, in particular, after repeat infusion of the immunotherapeutic agent. However, so far only few cases of severe allergic reactions or anaphylactic shock related to immunotherapeutics have been described in the literature [52]. If anaphylactic shock is suspected epinephrine and antihistamines should be administered immediately [53].

Given that all these differential diagnoses have a clinical presentation that is very similar to CRS, making a definitive diagnosis of CRS is very challenging. Since some of the therapies given for conditions other than CRS can mitigate the effectiveness of immunotherapy, the development of reliable diagnostic test that help to make the diagnosis of CRS are a high priority for future research. Such tests could greatly improve the effectiveness and safety of CAR T cell therapy.

### Pathophysiology of CRS

**Fig. 2:**
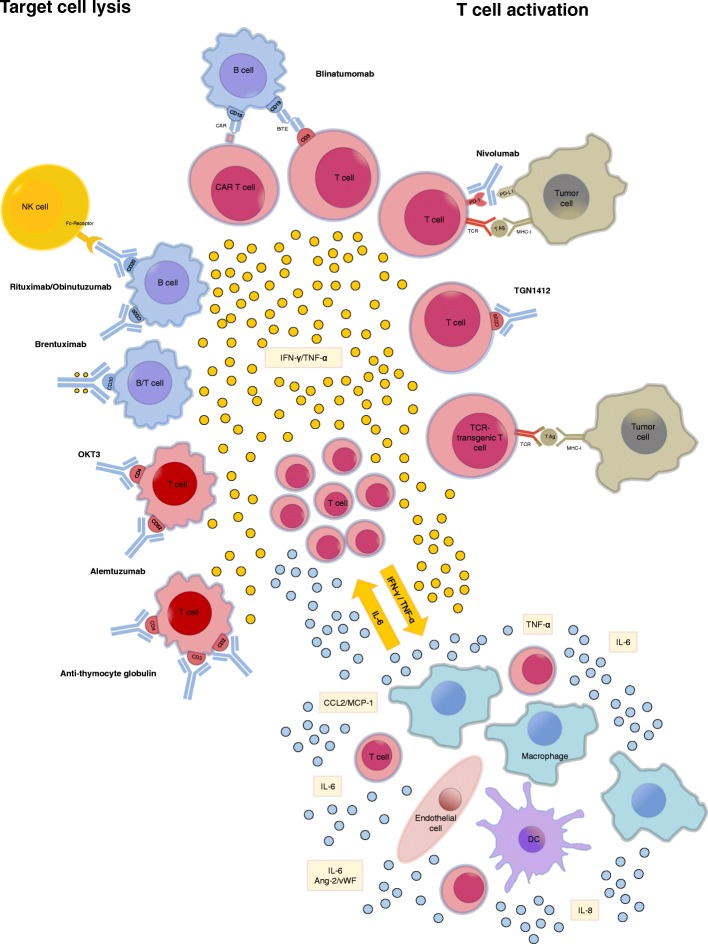
Reported inducers of CRS. CRS can be induced by direct target cell lysis with consecutive release of cytokines like interferon gamma (IFN-γ) or tumor necrosis factor alpha (TNF-α) or by activation of T cells due to therapeutic stimuli with subsequent cytokine release. These cytokines trigger a chain reaction due to the activation of innate immune cells like macrophages and endothelial cells with further cytokine release. Abbreviations: Ang-2: Angiopoetin 2; CAR: chimeric antigen receptor; DC: dendritic cell; IFN-γ: interferon gamma; MHC-I: major histocompatibility complex I;NK cell: natural killer cell; PD-(L)1: programmed cell death protein (ligand) 1; TCR: T cell receptor.; TNF-α: tumor necrosis factor alpha; vWF: von Willebrand factor

The pathophysiology of CRS is only incompletely understood. CRS is usually due to on-target effects induced by binding of the bispecific antibody or CAR T cell receptor to its antigen and subsequent activation of bystander immune cells and non-immune cells, such as endothelial cells. Activation of the bystander cells results in the massive release of a range of cytokines. We know little about how the initial activation of CAR T cells results in the distortion of the cytokine network that drives the inflammatory process in CRS. Depending on a number of characteristics of the host, the tumor, and the therapeutic agent the administration of T cell-engaging therapies can set off an inflammatory circuit that overwhelms counter-regulatory homeostatic mechanisms and results in a cytokine storm that can have detrimental effects on the patient. Figure 2 summarizes our current understanding of the pathophysiology of CRS.

IL-6, IL-10, and interferon (IFN)-Υ are among the core cytokines that are consistently found to be elevated in serum of patients with CRS. In the setting of T cell-engaging therapies, CRS is triggered by the massive release of IFN-γ by activated T cells or the tumor cells themselves. IFN-γ causes fever, chills, headache, dizziness, and fatigue. Secreted IFN-γ induces activation of other immune cells, most importantly macrophages [54]. The activated macrophages produce excessive amounts of additional cytokines such as IL-6, TNF-α, and IL-10. TNF-α elicits flu-like symptoms similar to IFN-γ with fever, general malaise, and fatigue but furthermore is responsible for watery diarrhea, vascular leakage, cardiomyopathy, lung injury, and the synthesis of acute phase proteins.

Interleukin 6 (IL-6) seems to hold a key role in CRS pathophysiology since highly elevated IL-6 levels are seen in patients with CRS [5, 55–57] and in murine models of the disease [58]. IL-6 can signal via two different modes. Classical IL-6 signaling involves binding of IL-6 to membrane-bound IL-6 receptor. Of note, the IL-6 receptor does not possess intracellular signaling domains. Instead, upon binding of soluble IL-6 to membrane-bound IL-6 receptors, the IL-6/IL-6 receptor complex binds to membrane-bound gp130, which initiates signaling through its intracellular domain. In trans-signaling, IL-6 binds to the soluble form of the IL-6 receptor, which has been cleaved from the cell surface by metalloproteinases. This soluble IL-6/IL-6 receptor complex binds to gp130 and therefore can also induce signaling in cell types that do not express membrane bound IL-6 receptors [59].

IL-6 contributes to many of the key symptoms of CRS. Via trans-signaling IL-6 leads to characteristic symptoms of severe CRS, i.e. vascular leakage, and activation of the complement and coagulation cascade inducing disseminated intravascular coagulation (DIC) [57, 60]. In addition, IL-6 likely contributes to cardiomyopathy that is often observed in patients with CRS by promoting myocardial dysfunction [61].

Recently, Teachey et al. performed a screen for biomarkers in patients after CAR T cell therapy for ALL and found that peak levels of IL-6, soluble IL-6 receptor, IFN-γ, and sgp130 correlated with the risk of severe CRS in a cohort of 35 pediatric and adult B-ALL patients receiving CD19-CAR T cell therapy. They subsequently validated these findings in 12 pediatric patients [62]. While limited due to the relatively small number of patients experiencing sCRS and limited availability of on-site cytokine measurement these tools might help in identifying patients that need a more intense monitoring and treatment.

A hallmark of severe CRS seems to be the activation of endothelial cells. Typical marker of endothelial activation such as Ang-2 and von Willebrand factor are often elevated in the serum of patients with CRS [26]. This indicates that the endothelium plays an important role in the pathophysiology of CRS both by amplifying the inflammatory response and as a target organ. The crucial contribution of endothelial dysfunction in the pathogenesis of CRS provides an explanation for some of the hallmarks of severe CRS, i.e. capillary leakage, hypotension, and coagulopathy [26]. As shown by a recent post mortem study in a patient who died of CRS after CD19-targeted CAR T cell therapy, endothelial cells seem to be an important source of IL-6 in severe CRS [63]. Importantly, endothelial activation and the ensuing vascular dysfunction might be the mechanistic factor linking CRS with neurotoxicity. A recent study found that neurotoxicity after immunotherapy with CD19-targeted CAR T cells was accompanied by findings consistent with endothelial activation [64].

In patients with CRS who develop a HLH/MAS-like syndrome additional cytokines such as IL-18, IL8, IP10, MCP1, MIG, and MIP1β are also elevated [62]. These cytokines also have been reported to be elevated in classical HLH and MAS. Why some patients develop HLH/MAS and others do not is poorly understood. Some patients may harbor genetic variants that predispose them to developing HLH/MAS. In addition, IL-6 may also promote the development of HLH/MAS in the setting of CRS by inducing dysfunction of cytotoxic activity in T and NK cells, which is a hallmark of HLH and MAS [65]. However, a link to genetic aberrations involved in the release of cytotoxic molecules (perforin, syntaxin) related to familial HLH (*PRF1*, *STX11*, *STXBP2,* and *MUNC13–4*) could not be established in a recent CAR T cell trial [55].

### Clinical management of CRS

**Fig. 3:**
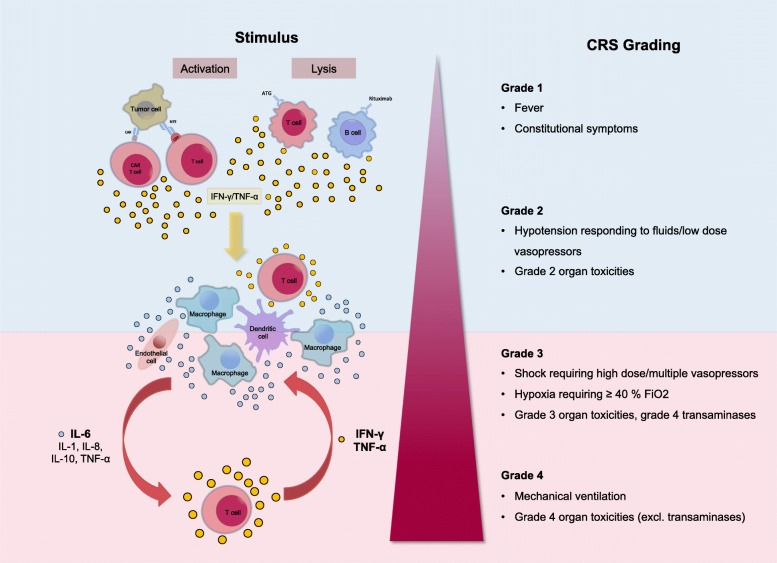
Proposed pathomechanism of CRS. Activation of manly T cells or lysis of immune cells induces a release of interferon gamma (IFN-γ) or tumor necrosis factor alpha (TNF-α). This leads to the activation of macrophages, dendritic cells, other immune cells and endothelial cells. These cells further release proinflammatory cytokines. Importantly, macrophages and endothelial cells produce large amounts of interleukin 6 (IL-6) which in a positive feedback loop manner activates T cells and other immune cells leading to a cytokine storm. Abbreviations: CAR: chimeric antigen receptor; FiO2: fraction of inspired oxygen; IL-6: interleukin 6; IFN-γ: interferon gamma; TNF-α: tumor necrosis factor alpha

The management of the toxicities of cancer immunotherapy is challenging clinical problem. Since T cell-engaging therapies are a relatively recent development there are still many unanswered questions regarding the optimal clinical management of CRS. The recommendations for the management of CRS are thus still evolving constantly. Current treatment algorithms for CRS are based on expert opinion and represent the experience of the pioneers in the field of T cell-engaging immunotherapies [28, 29]. The most widely used grading scheme for the severity of CRS was developed by the National Cancer Institute (NCI) (Fig. 3) [29].

Even though there are many commonalities regarding the clinical presentation and pathophysiology of CRS in patients receiving BiTE or CAR T cells, there are also important differences. The most important difference between BiTE and CAR T cells is that BiTE can be given repeatedly while CAR T cells are usually manufactured in limited amounts and thus are only administered once. The current approaches to prevention and treatment of CRS in patients receiving these two types of T cell-engaging therapies therefore differ substantially.

Effective management of patients that become critically ill following T cell-engaging therapy requires close collaboration between different specialties including hematology/oncology, neurology, radiology, and critical care. ICU referral should be considered in all patients with CRS and early involvement of the critical care team is paramount [66]. Since even severe CRS has a relatively good prognosis when appropriately treated, patients with CRS should be offered the full spectrum of modern critical care including mechanical ventilation if necessary [67].

The management of CRS follows a grade- and risk-adapted strategy for monitoring and therapy [28, 29]. Fever is an important clinical sign that should raise the suspicion of impending CRS in patients receiving T cell-engaging therapies. In the case of CAR T cell therapy, fever precedes the onset of CRS by at least 1 day and on average occurs 3 days prior to CRS. Therefore, patients who develop fever should be frequently reassessed for signs of CRS and outpatients should be admitted to hospital for closer observation [26]. Serum CRP has been suggested as a valuable biomarker for determining the severity of CRS [36]. However, by itself CRP seem insufficient to reliably predict the occurrence or severity of CRS [62]. Until better predictive biomarkers have been discovered, clinicians should therefore maintain a high alertness for the development of CRS in any patient following treatment with T cell-engaging therapies.

Low grade CRS is treated symptomatically with antihistamines, antipyretics and fluids. Additional diagnostic testing should be performed to rule out differential diagnoses. If an infection cannot be ruled out with certainty start of an empiric antibiotic therapy should be considered. Furthermore, all patients with early signs of CRS should be regularly evaluated for signs of further deterioration.

Severe CRS represents a life-threatening situation that requires prompt and aggressive treatment. With the BiTE blinatumomab the focus has been to prevent the occurrence of severe CRS. Based on the insight gained from the first clinical trials of blinatumomab a prophylactic protocol consisting of cytoreduction, dose adjustment, and premedication with corticosteroids has been devised, that resulted in a reduced incidence of severe CRS [68].

Unlike BiTEs, which can be given repeatedly, and can be interrupted if necessary, CAR T cell therapies are often administered only once and their final effective dose after in vivo expansion is unpredictable. Therefore, the primary aim in the setting of CAR T cell therapies so far has been to efficiently treat severe CRS once it develops while trying to avoid mitigation of the antitumoral immune response.

The observation that IL-6 is elevated in the serum of patients with CRS following CAR T cell therapy has led some investigators to consider tocilizumab for the treatment of severe CRS. IL-6 represents a particularly suitable target since IL-6 is of relatively little importance for T cell function [69, 70] but, as already mentioned, is a central driver of many symptoms of CRS. By binding to membrane-bound as well as soluble IL-6 receptor tocilizumab interferes with both classical and trans-signaling pathways. Subsequent studies confirmed that administration of monoclonal antibodies against IL-6 (siltuximab) and its receptor (tocilizumab) led to rapid resolution of CRS symptoms [29, 31, 33, 71]. In an early stage clinical trial tocilizumab demonstrated a 69% response rate in patients with severe or life-threatening CRS. As a consequence, tocilizumab has quickly become the gold standard for the initial treatment of severe CRS in patients receiving CAR T cells. In August 2017, concurrently with tisagenlecleucel the FDA approved tocilizumab for the treatment of CRS in patients 2 years of age or older. The recommended dose for i.v. application is 8 mg/kg body weight for adults and 12 mg/kg body weight for patients < 30 kg body weight up to a maximum of 800 mg per dose with an interval between consecutive doses of at least 8 h [72].

Patients that develop grade 3 or 4 CRS toxicity should immediately receive treatment with tocilizumab. Usually significant resolution of CRS related symptoms e.g. fever and hypotension is achieved within a few hours up to 2 days after the first application of tocilizumab. If no such effect is evident within 24 to 72 h a second administration is feasible. Of note, it should be taken into consideration that after administration of tocilizumab CRP can no longer be used as an indicator of CRS severity as blockade of IL-6 signaling results in a rapid decrease of CRP. Currently, there are several other IL-6-targeting monoclonal antibodies in late stage clinical development, which could also potentially be used to treat CRS. Siltuximab, is a chimeric, IGκ monoclonal antibody that binds human IL-6 and prevents it from interacting with both the membrane-bound and soluble form of the IL-6 receptor. Clazakizumab is another monoclonal antibody targeting IL-6.

Corticosteroids should generally be avoided as first line treatment of CRS in patients receiving CAR T cells and should be reserved for cases refractory to IL-6 blockade or patients with severe neurotoxicity. Since tocilizumab does not cross the blood-brain-barrier it does not seem to be very effective against CRES and corticosteroids are thought to be more effective than IL-6 targeting in this setting [29]. The current recommendations therefore prefer the use of corticosteroids for the treatment of the neurologic adverse effects of T cell-engaging therapies. Monoclonal antibodies that target IL-6 directly, thereby eliminating it from the circulation, might be advantageous in patients with severe CRS and concurrent neurotoxicity, since tocilizumab does not cross the blood brain barrier and therefore fails to inhibit IL-6 signaling in the CNS. Corticosteroids should also be considered in patients who develop HLHI/MAS as part of their CRS. If corticosteroids are used in patients receiving T cell-engaging immunotherapy, the duration of treatment should be kept as short as possible to minimize the detrimental effects on the effectiveness of immunotherapy.

In cases where neither tocilizumab nor glucocorticoids are effective blockade of TNF-α signaling has also shown effectiveness. However, a case of sCRS that was unresponsive to tocilizumab, etanercept, and glucocorticoids has been published [35]. In those cases other immunosuppressants, such as the IL-6 monoclonal antibody siltuximab [73], T cell-depleting antibody therapies such as alemtuzumab and ATG, IL-1R-based inhibitors (anakinra) or cyclophosphamide might be of benefit. Other experimental therapies for CRS include ibrutinib [74]. Moreover, there are several reports of successful use of cytokine adsorption in the setting of severe HLH unresponsive to standard treatment [75, 76] that might also be effective in sCRS.

The administration of tocilizumab does not appear not to negatively affect response rates to T cell-engaging therapies [39, 77–80]. Interestingly, in some studies even glucocorticoids did not seem to impact response rates [39, 78, 80]. However, in a study of 30 B-ALL patients treated with CD19-CAR T cells (CTL019) 2 of 9 patients treated with immunosuppression eventually relapsed with 11 patients relapsing overall within a follow-up of 2 to 24 months [32]. Similarly, in another B-ALL study high-dose steroids markedly reduced CAR T cell expansion in three cases with severe CRS and all patients experienced disease recurrence after CD19-targeted CAR T cell therapy [33]. In addition, smaller studies implicated a negative effect of glucocorticoid administration on patient outcome: In a case report of 3 patients suffering from chronic lymphocytic leukemia the only patient not completely responding to CD19-CAR T cell therapy received glucocorticoids for CRS treatment [40].

There are a variety of preventative measures that have the potential to reduce the incidence of CRS after immunotherapy [81]. One approach for mitigation of CRS risk is dose-reduction. Dose adaption of CAR T cells to tumor burden and the type of malignancy was shown to be effective in preventing severe CRS [35] and dose-reduced readministration of blinatumumab after a grade IV CRS was shown to be safe [82].

It can be expected that the treatment algorithms for CRS will change in the future as we gain more and more experience with managing the side effects of T cell-engaging immunotherapeutics. The effective treatment of CRS with greatly benefit from the ongoing efforts for harmonization of the grading system and treatment protocols for CRS.

### Future outlook

The CRS is among the most frequent serious adverse events and a represents a major cause of morbidity following T cell-engaging immunotherapy. Insights gained from studying the biological mechanisms of CRS and the clinical use of corticosteroids and IL-6 blockade have already improved the management of patients with CRS. However, there remain many unanswered questions and there still is amble room for improvement of the clinical management of CRS.

With the growing use of T cell-engaging therapies there is an urgent need for clinical trials that improve the evidence base for the treatment of CRS. Our current management strategies for CRS are predominantly based on biologic reasoning, expert opinion, and retrospective analyses. In order to further improve the safety and effectiveness of the clinical management of CRS, randomized controlled trials that evaluate different treatment strategies for CRS are necessary.

Furthermore, a primary challenge in the future management of CRS will be to identify additional targets for specific therapeutic intervention in CRS. Given the apparent central role of the endothelium in the pathophysiology of CRS and neurotoxicity following T cell-engaging therapies further studies on the role of endothelial dysregulation in CRS appear particularly promising and should provide valuable insight that could lead to novel therapeutic approaches.

The clinical management of CRS could further be improved by the identification of biomarkers that reliably predict the development of CRS [83]. We will need to develop tools that guide treating physicians in fine-tuning the use of pharmacologic agents that interfere with these targets in order to ameliorate the adverse effects of CRS while maintaining the therapeutic activity of T cell-engaging therapy.

Another promising line of research that will certainly help to improve the safety of T cell-engaging immunotherapy focuses on the optimization of the design of the compounds themselves [84]. Several research groups are focusing on the design of improved CAR constructs with high antitumor activity but a lower risk of CRS. Next generation CAR constructs will enable the conditional activation of CAR T cells or will include suicide switches that allow controlled depletion of CAR T cells [85, 86]. Such improvements in the construction of immunotherapies will likely result in a further reduction of serious adverse effects in the future.

## Conclusion

In the wake of the remarkable success of recently developed immunotherapies the field of immuno-oncology is poised to continue its rapid growth. As a consequence of the more widespread application of immunotherapeutic anticancer agents an increasing incidence of CRS cases can be expected in the upcoming years. A thorough understanding of the clinical presentation, underlying pathophysiology, and available therapeutic options as well as the most important differential diagnoses is crucial for the effective management of this clinical syndrome. Basic research findings and investigations of patients with CRS will provide valuable insight into the underlying mechanisms of CRS and could aid in the development of molecularly-targeted treatment strategies to prevent and treat CRS. The case of IL-6 blockade in CRS illustrates the potential of targeted immunological interventions for the management of toxicities of cancer immunotherapy. With improved understanding of the pathophysiology and increasing clinical experience in toxicity management more specific mitigation of the CRS will hopefully make cancer immunotherapy safer and more effective.
